# Automatic Respiratory Gating Hepatic DCEUS-based Dual-phase Multi-parametric Functional Perfusion Imaging using a Derivative Principal Component Analysis

**DOI:** 10.7150/thno.37284

**Published:** 2019-08-14

**Authors:** Diya Wang, Guy Cloutier, Yan Fan, Yanli Hou, Zhe Su, Qiang Su, Mingxi Wan

**Affiliations:** 1 Department of Biomedical Engineering, School of Life Science and Technology, Xi' an Jiaotong University, Xi' an, P. R. China.; 2 University of Montreal Hospital Research Center, Montreal, QC, Canada.; 3 Department of Radiology, Radio-Oncology and Nuclear Medicine, and Institute of Biomedical Engineering, University of Montreal, Montreal, QC, Canada.; 4 Alliance Franco-Chinoise, Montreal, QC, Canada.; 5 Department of Oncology, Beijing Friendship Hospital, Capital Medical University, Beijing, 1000050, P. R. China.

**Keywords:** contrast-enhanced ultrasound, functional perfusion imaging, respiratory motion, machine learning, principal component analysis

## Abstract

**Purpose**: Angiogenesis in liver cancers can be characterized by hepatic functional perfusion imaging (FPI) on the basis of dynamic contrast-enhanced ultrasound (DCEUS). However, accuracy is limited by breathing motion which results in out-of-plane image artifacts. Current hepatic FPI studies do not correct for these artifacts and lack the evaluation of correction accuracy. Thus, a hepatic DCEUS-based dual-phase multi-parametric FPI (DM-FPI) scheme using a derivative principal component analysis (PCA) respiratory gating is proposed to overcome these limitations.

**Materials and Methods**: By considering severe 3D out-of-plane respiratory motions, the proposed scheme's accuracy was verified with *in vitro* DCEUS experiments in a flow model mimicking a hepatic vein. The feasibility was further demonstrated by considering *in vivo* DCEUS measurements in normal rabbit livers, and hepatic cavernous hemangioma and hepatocellular carcinoma in patients. After respiratory kinetics was extracted through PCA of DCEUS sequences under free-breathing condition, dual-phase respiratory gating microbubble kinetics was identified by using a derivative PCA zero-crossing dual-phase detection, respectively. Six dual-phase hemodynamic parameters were estimated from the dual-phase microbubble kinetics and DM-FPI was then reconstructed via color-coding to quantify 2.5D angiogenic hemodynamic distribution for live tumors.

**Results**: Compared with no respiratory gating, the mean square error of respiratory gating DM-FPI decreased by 1893.9 ± 965.4 (*p* < 0.05), and mean noise coefficients decreased by 17.5 ± 7.1 (*p* < 0.05), whereas correlation coefficients improved by 0.4 ± 0.2 (*p* < 0.01). DM-FPI observably removed severe respiratory motion artifacts on PFI and markedly enhanced the accuracy and robustness both *in vitro* and in *vivo*.

**Conclusions**: DM-FPI precisely characterized and distinguished the heterogeneous angiogenic hemodynamics about perfusion volume, blood flow and flow rate within two anatomical sections in the normal liver, and in benign and malignant hepatic tumors. DCEUS-based DM-FPI scheme might be a useful tool to help clinicians diagnose and provide suitable therapies for liver tumors.

## Introduction

Liver cancer is respectively the second and sixth major causes of death in developing and developed countries 1. Angiogenesis from the existing hepatic branch arterioles and veins holds a key role in the fast expansion of hepatocarcinoma cells, through nutrient feeding from normal tissues 2-5. Angiogenic perfusion is thus of importance to identify tumors and grade their stages 4, 5. For this purpose, dynamic contrast-enhanced ultrasound (DCEUS) using microbubble contrast agents have been proven of interest in angiogenic perfusion monitoring for liver tumors 5-9.

In comparison with several perfusion imaging techniques based on computerized tomography and nuclear magnetic spin imaging, DCEUS is a more low-cost, real-time, and non-irradiating technique for the easy bedside use 10-12. Backscattered echoes in blood are initially heightened through microbubble injection in DCEUS 13. With similar sizes with red blood cells, these microbubbles show up strong nonlinear scattering under insonation 13. These microbubbles' transient, linear, and nonlinear backscattered echoes are then developed by using DCEUS technologies 14, 15. These specific techniques aim to control backscattered echoes of tissue, motivate microbubble signals, and enhance discrimination of microvessels 14, 15. DCEUS has thus been widely utilized to monitor microvascular spreading and their microcirculation during tumor growth, especially in hepatic tumors 12, 16, 17.

Among quantitative DCEUS techniques, functional perfusion imaging (FPI) based on bolus detected kinetics was proposed to quantify detailed hemodynamics in angiogenesis 8, 9, 18. The homodynamic parameters related with time, intensity, and ratio were calculated from microbubble-enhanced time-intensity curve (TIC) to develop multi-parametric FPI technique 19-21. These parameters are in direct proportion to the blood flow, perfusion volume, and flow rate, respectively 8, 12, 21. Angiogenic perfusion using DCEUS-based FPI could characterize hepatic cirrhosis 22, 23, nodular hyperplasia 7, 17, hemangioma 7, 17, hepatocellular carcinoma 22, 24, and other metastatic cancers 8, 16.

Conventional FPI depicts and quantifies the 2D hemodynamics in angiogenesis at only one anatomical section without 3D perfusion information 8, 18. Moreover, the accuracy and robustness of conventional FPI are seriously limited by tissue deformation and respiratory motion artifacts 19, 25. The respiration causes out-of-plane motion affecting the characterization of angiogenesis and may contort the shape, dimension, and position of tumors identified by FPI 17. These disturbances not only decrease the TICs' signal-to-clutter ratio (SCR) 26 but also severely distort hepatic DCEUS-based FPI due to regions-of-interest (ROIs) misalignment and TIC kinetics misregistration 19, 27.

To weaken the influence of respiration on abdominal DCEUS, breath-holding is still used for short examinations. However, it is infeasible for long perfusion imaging (up to several minutes), especially for patients with cardiopulmonary diseases 19, 28. Thus, various respiratory motion compensation strategies have been investigated for abdominal DCEUS studies with free-breathing 16, 17, 19, 27. External sensor and tracking systems were used to capture DCEUS images in the same breathing phase 29. However, these systems enhance the duration of examinations, which is often impracticable clinically 28. As an alternative, semi-automatic 2D and 3D registration methods were developed based on images 7, 16, 17. However, these registration strategies were always initialized by a manual drawing of ROI, of which motion tracking is controversial in handling out-of-plane frames and unavailable anatomical landmarks 7, 17.

To overcome limitations of abovementioned compensation strategies, semiautomatic and fully automatic respiratory gating methods employing machine learning algorithms have been developed in recent years. Using independent component analysis, a posteriori respiratory gating DCEUS approach was proposed based on an assumption of independence between microbubble kinetics and breathing motion 28. Whereas, the assumption could not be confirmed 7, 17, and the reliability of the respiratory gating strategy for DCEUS-based FPI could not be guaranteed 19. A principal component analysis (PCA) was implemented on respiratory kinetics in the framework of DCEUS for focal liver lesion assessment 7, 30. Although orthonormal components in PCA of respiratory kinetics 27 overcome the dubious independence assumption in the independent component analysis 19, the use of PCA is still limited by the lack of validation of FPI and a breathing rate foreknowledge 27 and tumor location 7. In some cases, some orthonormal components in PCA are negative. Hemodynamics and breathing curves can thus be taken as a problem of non-negative matrix factorization to ensure valid respiratory motion compensation in abdominal DCEUS studies 19, 31. But the non-negative assumption may be unnecessary for a correct respiratory gating.

*In vitro* and *in vivo* abdominal DCEUS-based FPI of a single respiratory phase was reconstructed by considering slight out-of-plane motion 19, 32. 1D perfusion quantification was also corrected under an extreme condition of out-of-plane motion in our previous study 33. However, to our knowledge, no study has been conducted on 2D DCEUS-based FPI with serious distortion of angiogenesis under a condition of extreme out-of-plane breathing motion. Out-of-plane motion results in severe image artifacts which inevitably exist in free-breathing hepatic DCEUS studies 19, 32. Under this condition, abovementioned compensation strategies are meaningless and the gating strategies have not been proven to be reliable in the context of DCEUS-based FPI. Moreover, the accuracy of *in vivo* respiratory gating FPI is unknown because of the uncontrolled DCEUS without respiratory motion and the unknown ground truth of* in vivo* respiratory curves 7, 27, 28. Therefore, a hepatic DCEUS-based dual-phase multi-parametric FPI (DM-FPI) scheme using derivative PCA respiratory gating is proposed in this study to characterize and quantify angiogenic hemodynamics in hepatic tumors. The accuracy and feasibility of the proposed scheme were respectively illustrated through *in vitro* and *in vivo* hepatic perfusion examinations under the condition of extreme out-of-plane respiratory motion. A list of abbreviations used in this study is given in Table 1.

## Materials and Methods

### *In vitro* Experiments

An ultrasound scanner (#G50, Vinno Inc., Suzhou, China) utilizing a linear transducer was applied for *in vitro* perfusion experiments (n = 20) to validate the accuracy of the proposed respiratory gating DM-FPI scheme. Table 2 displays the experimental parameter settings. A wide dynamic range was used to avoid saturation of DCEUS sequences which enabled the valid reconstruction of DM-FPI 34. The transducer was attached onto a controllable 3D rotary motion system to produce out-of-plane motion and to record backscatter echoes from a mimicked hepatic vein (diameter and wall thickness: 5 and 1.5 mm; Shuguang, Xi'an, China). A 0.9% normal saline solution was circulated in the flow phantom with a mean flow of 10 ml/s controlled by a flexible tubepump (#BT300-1F, Longer, Baoding, China). A bolus of 0.2 mL diluent of microbubbles (2×10^5^ bubbles/mL; SonoVue, Bracco, Milan, Italy), which were made of phospholipid encapsulated sulfur hexafluoride, was injected via an infusion outfit into the flow phantom. The transducer's 3D rotary motion was regulated by an electronic control system (#MC600, PSA-11AS, Zolix, Beijing, China) to simulate out-of-plane breathing motion and to result in an extreme 3D periodic rotational motion in DCEUS loops. Thus, DCEUS loops exhibited an serious deformation of the mimicked hepatic vein with a simulated respiratory frequency of 0.27 Hz 35. Pulse-inversion contrast harmonic imaging technique 14, 15 was used to record all *in vitro* DCEUS loops with respiratory motion artifacts. Moreover, *in vitro* static DCEUS loops without breathing motion artifacts at fixed end-of-expiration (EOE) and end-of-inspiration (EOI) phases were respectively recorded as ground truths to assess the respiratory gating accuracy in the proposed DM-FPI scheme.

### *In vivo* Experiments

An ultrasound platform (#DC-8, Mindray Inc., Shenzhen, China) using a convex array was utilized for *in vivo* hepatic (n = 14) perfusion experiments to validate the feasibility of the proposed respiratory gating DM-FPI scheme. Experimental acquisitions included 8 healthy rabbits, 6 patients with diagnosed symptoms of hepatic cavernous hemangioma (HCH, n = 3) and hepatocellular carcinoma (HCC, n = 3, one with a leak). Patients (2 females and 4 males) were 45 to 69 years old. As performed in the previous studies 19, 20, the normal hepatic perfusion experiments were conducted in healthy rabbits instead of humans because of ethical restrictions.

After the rabbits were anesthetized with a 3% concentration of mebumalnatrium, a bolus of SonoVue diluent (0.2 ‒ 0.5 mL, 2 × 10^8^ bubbles/mL) was intravenously injected 19, 20. The patients were asked to breathe freely and a bolus of SonoVue diluent (2.5 mL, 2 × 10^8^ bubbles/mL) was also intravenously injected and then immediately washed with 0.9% normal saline 8, 19. DCEUS sequences of patients were recorded in local hospitals. The Local Research Ethics Committee approved this study. The consent forms of patients were obtained before the examination. All *in vivo* DCEUS loops with respiratory motion artifacts were also recorded using the same pulse-inversion contrast harmonic imaging mode. All *in vitro* and* in vivo* DCEUS sequences covered the major perfusion phases including pre-contrast, arriving, rising, filling, and complete or part falling phases of microbubbles.

### Respiratory Gating DCEUS-based Dual-phase Multi-parametric FPI

**Figure 1:**
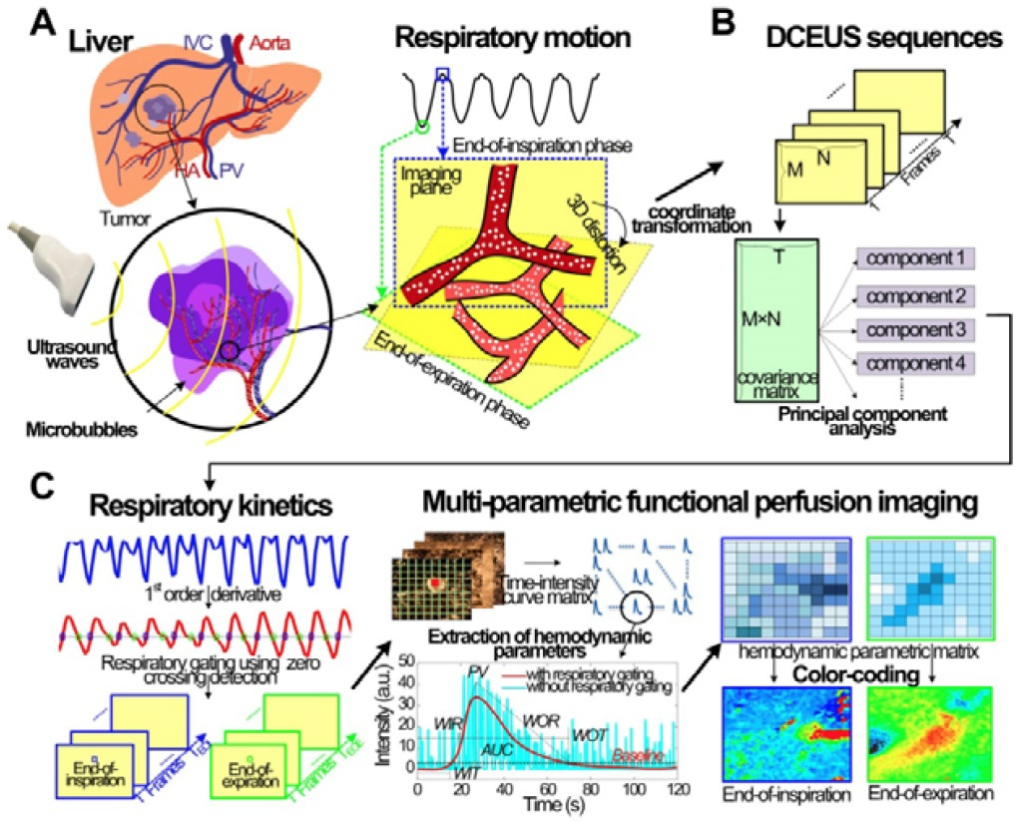
(Color online) Schematic diagram of automatic respiratory gating hepatic DCEUS-based DM-FPI. (a) Schematic of 3D deformation of hepatic angiogenesis caused by out-of-plane breathing motion in DCEUS loops, (b) respiratory kinetics estimated by using PCA, (c) respiratory gating DCEUS-based DM-FPI by using derivative PCA. DCEUS = dynamic contrast-enhanced ultrasound, DM-FPI = dual-phase multi-parametric functional perfusion imaging, PCA = principal component analysis.

Figure 1 shows the schematic diagram of the proposed automatic respiratory gating hepatic DM-FPI based on DCEUS scheme under out-of-plane artifacts induced by free-breathing. Respiratory kinetics induced by free-breathing was first extracted using the second component of PCA from DCEUS sequences. Using derivative PCA zero-crossing dual-phase detection of the respiratory kinetics, Dual-phase TICs at EOI and EOE phases were then acquired from the corresponding dual-phase respiratory gating DCEUS subsequences, respectively. The respiratory gating hepatic DCEUS-based DM-FPI was finally reconstructed after color-coding of dual-phase three styles of six hemodynamic parameters estimated from the corresponding dual-phase TICs.

Hepatic DCEUS sequences can be orthogonally linearly transformed to a new coordinate system via PCA. With this method, the greatest and the second variances of its projection respectively correspond on the first coordinate and the second orthogonal coordinate 36. As well, PCA decomposition is used for dimensionality reduction in machine learning 36. Thus, the 3D DCEUS sequence 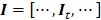 was first orthogonally linearly transformed as a 2D matrix 
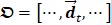
 at each pixel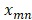.


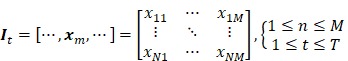
 (1)


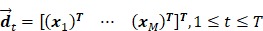
 (2)

where each column vector in 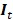 is 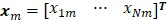
**.** *T*, *M*, and *N* are the numbers of frame, column, and row in ***I***, respectively. Each 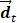 in 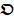 is a dynamic intensity change of all pixels in one frame at each time point *t*. Each 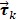 in 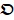 is a dynamic fluctuations of each point in 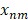 during the whole perfusion cycle *T*, which can be taken as TIC of each pixel.


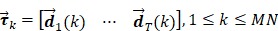
 (3)

The alteration in 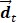 could be taken as a linear combination of spatial weighting matrix 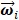 and principal component vectors 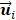 in Eq. (3) 33.


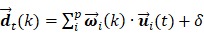
 (4)

where* p* is the component order,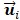 represents the *i*-th vector of principal component in 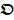
33. 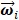 is the spatial weighting vector of 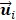 and is quantified by using the maximal eigenvectors from the covariance 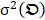 33. While *p* is larger than 4, the remanent component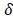 in the variance is smaller than 1.7% in 37. So *p* is 4 in this study 33. In terms of a foreknowledge of the previous validations 8, 27, 37, the second principal component 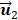 corresponds to the respiratory kinetic curve 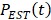, which can be estimated as:


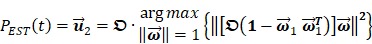
 (5)

Abnormal fluctuations and noise on raw respiratory kinetic curve 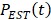 were first removed by smoothing to obtain 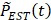. Zero-crossing dual-phase detection was applied on the first-order derivative curve of 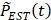 to alternately identify the two extreme phases of EOI (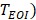 and EOE 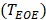 in the ascending and descending slopes in each respiratory cycle, as defined by the following equation:


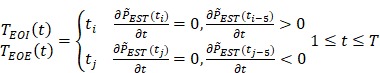
 (6)

As shown in Fig. 1c, the corresponding dual-phase DCEUS subsequences then formed at the same phases of 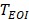 and 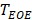, respectively. Imaging region of two DCEUS subsequences at 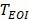 and 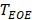 was respectively divided into small equal-sized ROIs of 3×3 pixels. TICs with respiratory gating (TIC_RG_) were then extracted pixel-by-pixel 12 by averaging the intensities of dual-phase DCEUS echoes within those ROIs at each time point. A dual-weighted moving average filter 14 was then applied in TIC_RG_ denoising to remove the clutter originated from the thermal noise and speckle effect 8 and to partly suppress recirculation noise and recover later phase 38. Thus, the dual-phase perfusion parameter estimation from TIC_RG_ was guaranteed.

As depicted in Fig. 1c, six hepatic hemodynamic-related perfusion parameters were calculated from the dual-phase TIC_RG_ for each group data. Two time parameters are wash-in time (WIT) and wash-out time (WOT), which are proportional to the blood flow; two intensity parameters are peak value (PV) and area under curve (AUC), which are related to the local perfusion volume; and two ratio parameters are wash-in rate (WIR) and wash-out-rate (WOR), which are in direct proportion to flow rate 8, 12, 21. More details and calculations of these perfusion parameters were introduced in 8, 32. Each perfusion parameter was normalized and then encoded in 256 levels as a standard RGB coding rule 8, 32 to reconstruct the corresponding perfusion map. Thus, respiratory gating hepatic DCEUS-based DM-FPI was described by using the color quantification map of above parameters.

### Data Processing

*In vitro* and* in vivo* FPIs without respiratory gating (FPI_noRG_) were reconstructed from TICs without respiratory gating (TIC_noRG_) to compare with respiratory gating FPI (FPI_RG_) of the same perfusion parameter. TIC_noRG_ was acquired from the same DCEUS loops used for TIC_RG_ and FPI_RG_ but without respiratory gating. Moreover, without breathing motion disturbances,* in vitro* static TIC (TIC_static_) and FPI (FPI_static_) were considered as the control groups to assess the accuracy of the proposed respiratory gating DM-FPI scheme. TIC_static_ was extracted from the ROI at the same location as the *in vitro* static DCEUS sequences. Additionally, FPI_RG_, FPI_noRG_, and FPI_static_ of the same hemodynamic parameters were adjusted to the same dynamic ranges to demonstrate the performances of the DM-FPI scheme 8.

The respiratory gating veracity of TIC_RG_ was evaluated by utilizing the SCR 26 and the mean-square-error (MSE) between TIC_RG_ and TIC_static_, as defined by the following equations 32:


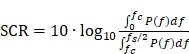
 (7)


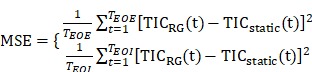
 (8)

where *P*(*f*) is the TIC power spectral density. *f_c_* and *f_s_* are the cut off and sample frequencies of TIC, respectively. MSE between TIC_noRG_ and TIC_static_ was also calculated to compare with the MSE between TIC_RG_ and TIC_static_.

The accuracy of respiratory gating DM-FPI was quantified by utilizing the MSE between FPI_RG_ and FPI_static_ and the mean correlation coefficients (R) between FPI_RG_ and FPI_static_ in the entire perfusion region, as defined by the following equations 32:


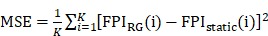
 (9)


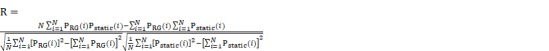
 (10)

where P_RG_ and P_static_ are the probability densities in FPI_RG_ and FPI_static_ of one perfusion parameter, respectively, *K* is the number of FPI, and *N* is the points of P_RG_ and P_static_. The MSE and correlation R between FPI_noRG_ and FPI_static_ were also computed to compare with the MSE and R between FPI_RG_ and FPI_static_.

The respiratory gating feasibility in *in vivo* hepatic TICs and DM-FPI were evaluated using the SCR of TICs and mean noise coefficients (MNC) of FPI in the entire perfusion region, as defined by the following equations:


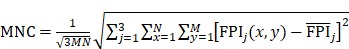
 (11)

where FPI*_j_* is the red, green, and blue components (*j* = 1, 2, 3) in the color map of FPI. 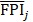
is the mean value of FPI*_j_*.

### Statistical Analysis

Mean values of perfusion results and their standard deviations were calculated and further evaluated using a t-test. Herein, a *p*-value < 0.01 was taken for highly significant that was marked by two asterisks in Figures and Tables. A *p*-value < 0.05 was regarded as statistically significant which were marked by one asterisk in Figures and Tables. Since three types of six FPIs were reconstructed at single EOE or EOI phase for each group of data, the analysis databases of *in vitro*, *in vivo* normal, HCH, and HCC data were 40, 16, 6, and 6 for each PFI type at single phase, respectively; the corresponding databases were 240, 96, 36, and 36 for of all types of dual-phase FPIs, respectively. All data processing was executed utilizing MATLAB (#2014a, MathWorks Inc., Natick, MA). Machine learning toolbox of MATLAB was used to extract the respiratory curves, which included the packages of non-negative matrix factorization, independent component analysis, and PCA.

## Results

### Respiratory Kinetics and Dual-phase Detection

**Figure 2:**
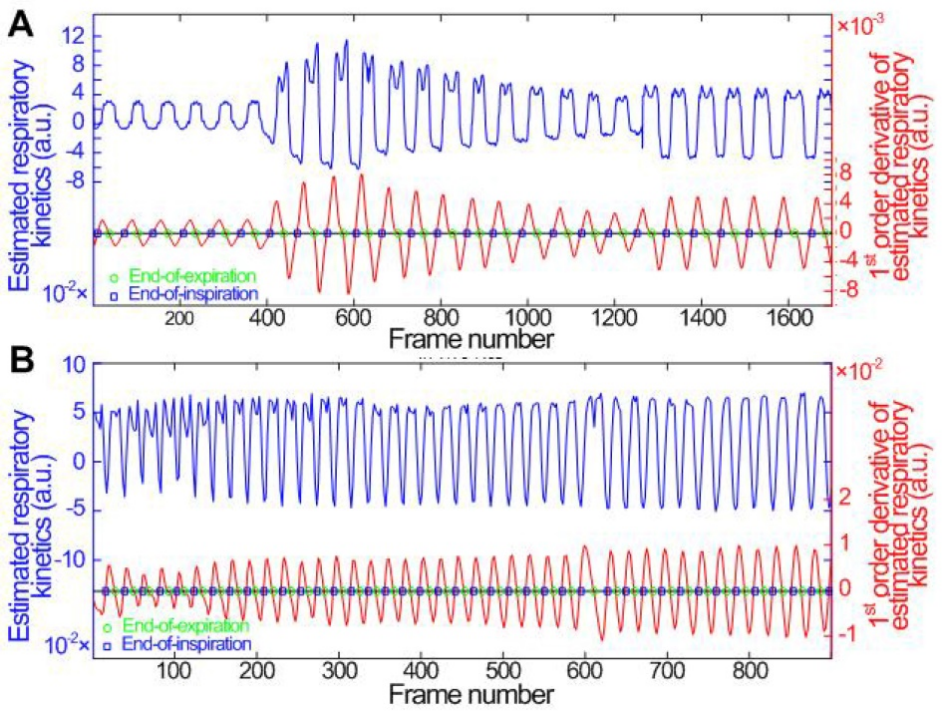
(Color online) Comparisons of (a) *in vitro* and (b) *in vivo* hepatic respiratory kinetics estimated by using PCA (blue lines) and derivative PCA (red lines), which automatically identified dual-phase DCEUS subsequences at EOE and EOI phases via zero-crossing dual-phase detection. EOE = end-of-expiration and EOI = end-of-inspiration.

**Figure 3:**
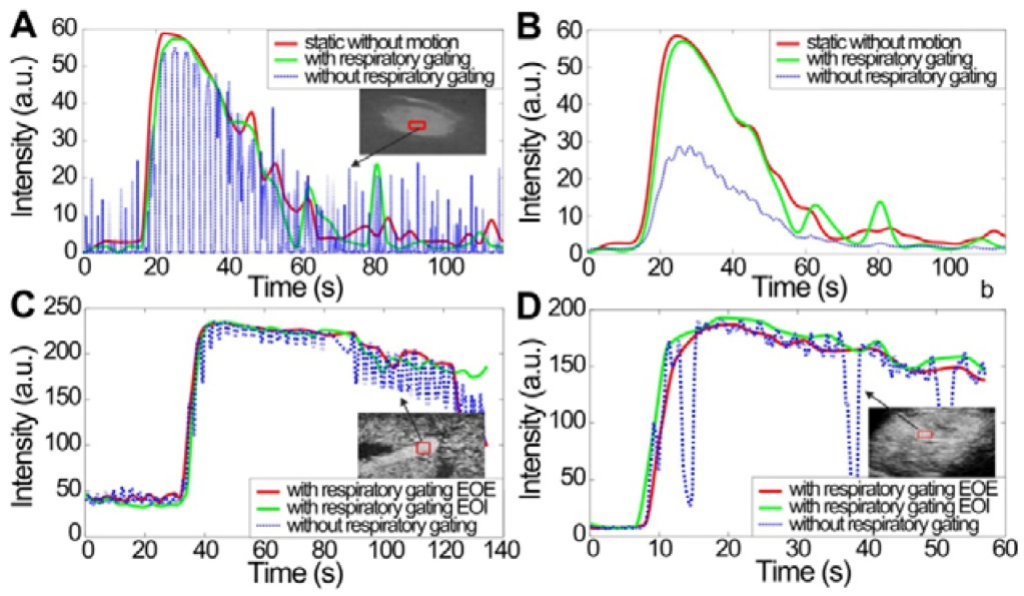
(Color online) Comparison of hepatic microbubble-enhanced TICs without breathing motion artifacts, and TICs without and with respiratory gating using derivative PCA zero-crossing dual-phase detection shown in Fig. 2. *In vitro* TICs (a) before and (b) after fitting, *in vivo* TICs at EOE and EOI phases extracted from (c) a normal hepatic vein and (d) a hepatic cavernous hemangioma. TICs = time-intensity curves.

Figures 2a and 2b show the *in vitro* and *in vivo* hepatic respiratory kinetic curves, which acquired from DCEUS loops in contrast mode and fundamental mode, respectively. The order of principal component vector in 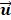 is related to reduction dimension 36, which is analogical with non-negative matrix factorization and independent component analysis 19, 31. Previous studies have demonstrated that the 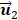 well matched the respiratory motion frequency estimated from the manual frame selection in respiratory gating methods, which were further verified in the cross-validations using non-negative matrix factorization and independent component analysis 19, 27, 37. The estimated frequency in 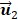 was 0.27 Hz in keeping with the simulated ground truth and the results in 35. Moreover, the estimated breathing phases in 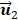 were also consistent with the simulated kinetic phases. Therefore, the respiratory kinetics was estimated by using the second principal component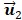 in this study. The estimated respiratory kinetics accounted for 20.3% ± 2.9 % of all components when *p* was 4. In comparison with *in vivo* respiratory kinetics, *in vitro* kinetics contained some information on microbubble kinetics, and the envelope was equivalent to TIC_RG_ (see Fig. 3a). This finding illustrates that the component was dependent in the extracted breathing kinetics, which verified the uncertain independence assumption of the independent component analysis between microbubble-enhanced curves and breathing kinetics in DCEUS studies 7, 17.

These estimated respiratory kinetic curves were smoothed and the EOE and EOI phases of DCEUS loops were automatically identified via the derivative PCA zero-crossing dual-phase detection illustrated in Figs. 2a and 2b. According to EOE and EOI phases, the corresponding dual-phase hepatic DCEUS subsequences were automatically selected from raw DCEUS loops, respectively. The mimicked and *in vivo* hepatic veins displayed complex 3D deformation due to the serious out-of-plane artifacts caused by breathing motion. The vertical and horizontal positions and axial and lateral dimensions of the mimicked hepatic vein were altered periodically at the EOE and EOI phases in the flow model. The gallbladder without perfusion and the right and left hepatic veins with a bolus injection of microbubbles appeared alternately at the EOE and EOI phases.

### Contrast-enhanced Dual-phase TICs with Respiratory Gating

**Figure 4:**
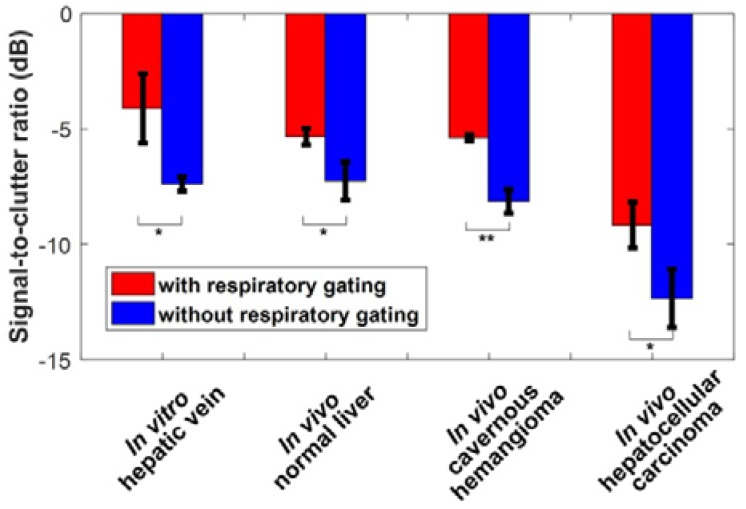
(Color online) Signal-to-clutter ratio of *in vitro* and *in vivo* hepatic TICs with and without respiratory gating using derivative PCA zero-crossing dual-phase detection.

Figure 3 shows *in vitro* and *in vivo* hepatic dual-phase TIC_RG_ and TIC_noRG_ that were obtained from the dual-phase DCEUS subsequences. *In vitro* dual-phase TIC_static_ was also acquired from two static DCEUS loops without respiratory motion artifacts at two same EOE and EOI phases. ROIs for TIC computations are indicated by red rectangles with a size of 20 × 40 pixels in the partial DCEUS images of Fig. 3. Figure 3b illustrates the *in vitro* filtered TICs. The hemodynamic characterizations of TIC_RG_ were consistent with the ground truth TIC_static_ results, and completely different from those of TIC_noRG_. In comparison with TIC_RG_, TIC_noRG_ was disturbed by obvious aperiodic and periodic fluctuations caused by the out-of-plane breathing motion. These oscillations produced misregistration of TIC kinetics, wrong estimation of hemodynamic parameters describing the hepatic angiogenesis, and distortion of hepatic DCEUS-based FPI 19, 27. Figure 4 illustrates the SCR of *in vitro* and *in vivo* hepatic TIC_RG_ and TIC_noRG_. In comparison with *in vitro* TIC_noRG_, the average MSE of *in vitro* TIC_RG_ reduced by 300.5 ± 19.8 (*p* < 0.01) 33. But the average SCR of *in vitro* and *in vivo* TIC_RG_ enhanced by 3.3 ± 1.7 (*p* < 0.05) 33 and 2.6 ± 0.7 dB (*p* < 0.05), respectively, when compared with the corresponding TIC_noRG_.

### *In vitro* DCEUS-based Dual-phase Multi-parametric FPI with Respiratory Gating

**Figure 5:**
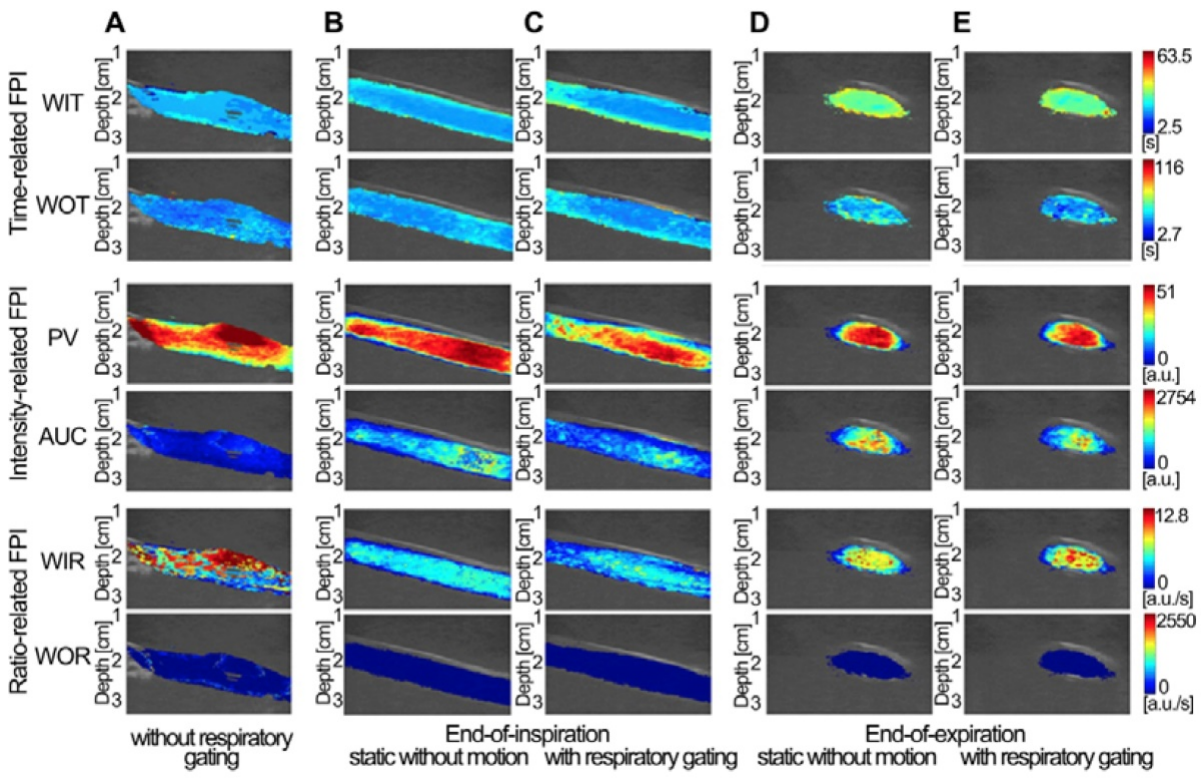
(Color online) Comparisons of* in vitro* DCEUS-based DM-FPI of the mimicked hepatic vein. (a) time-, intensity-, and ratio-related FPIs without respiratory gating, the corresponding static FPIs without respiratory motion artifacts at (b) the EOI and (d) EOE phases, and the corresponding respiratory gating FPIs at (c) the EOI and (e) EOE phases. WIT = wash-in time, WOT = wash-out time, PV = peak value, AUC = area under curve, WIR = wash-in rate, and WOR = wash-out rate.

**Figure 6:**
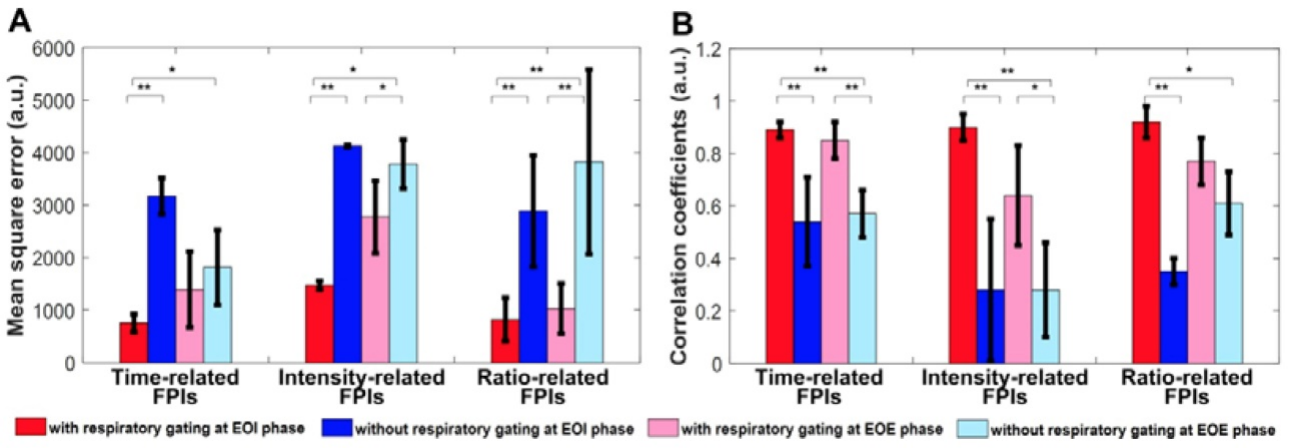
(Color online) Mean square errors and correlation coefficients of *in vitro* hepatic DCEUS-based DM-FPI without and with respiratory gating using derivative PCA zero-crossing dual-phase detection.

Three *in vitro* groups of dual-phase FPI_static_, FPI_RG_, and FPI_noRG_ of the mimicked hepatic vein were shown in Fig. 5. Each group included two time-related DM-FPIs of WIT and WOT, two intensity-related DM-FPIs of PV and AUC, and two ratio-related DM-FPIs of WIR and WOR. In comparison with changes in FPI_noRG_, inhomogeneous transient variations in blood velocity, perfusion charge, and rate of flow of the mimicked hepatic vein hemodynamics were clearly presented on the time-, intensity-, and ratio-related dual-phase FPI_RG_, respectively. Differences in the corresponding hemodynamic distributions depicted by dual-phase FPI_RG_ were also clearly observed at the EOI and EOE phases. Dual-phase FPI_RG_ in Figs. 5c and 5e was identified with the corresponding ground truths in FPI_static_ in Figs. 5b and 5d. *In vitro* FPI_RG_ was nearly similar to the ground truth. The inhomogeneous hemodynamic mappings at various respiratory phases were overlapped and distorted in FPI_noRG_ because of the absence of respiratory gating. As shown in Fig. 6, the accuracy in respiratory gating DM-FPI was directly quantified by utilizing the MSE and R of FPI_RG_ and FPI_noRG_ at EOE and EOI phases, respectively. Table 3 displays the decrease in MNC and the increase in correlation R of time-, intensity-, and ratio-related FPI_RG_ when compared with FPI_noRG_ in Fig. 6. In comparison with FPI_noRG_, the MSE of FPI_RG_ decreased significantly by 1893.9 ± 965.4 (*p* < 0.05) whereas the correlation R improved significantly by 0.4 ± 0.2 (*p* < 0.01).

### *In vivo* DCEUS-based Dual-phase Multi-parametric FPI with Respiratory Gating

**Figure 7:**
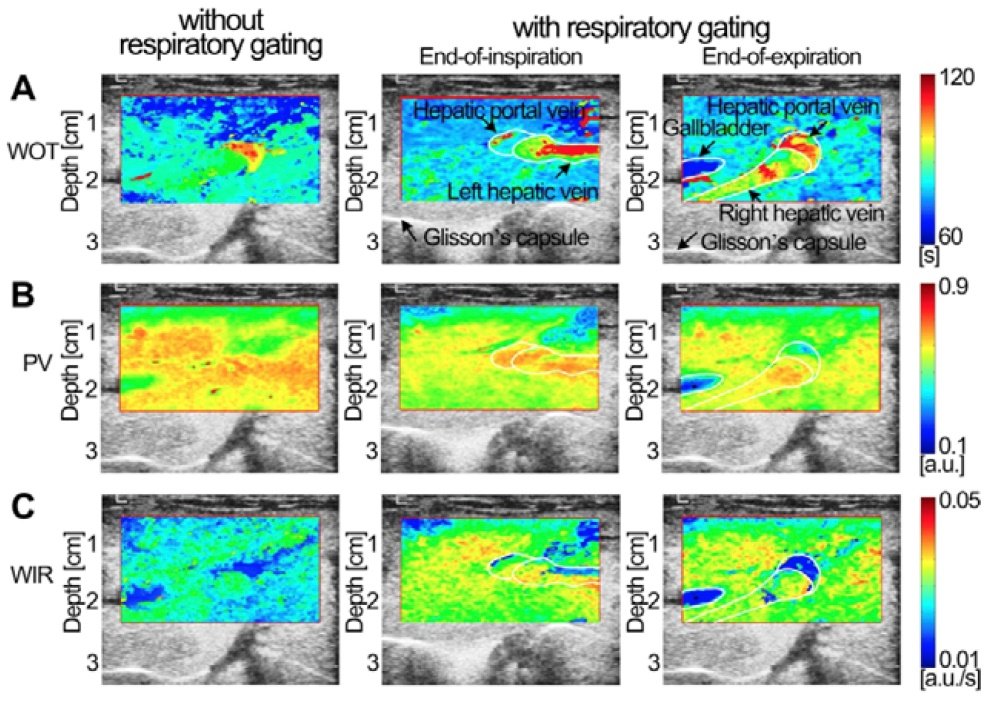
(Color online) Comparisons of *in vivo* normal hepatic DCEUS-based DM-FPI of a healthy rabbit. (a) A time-related FPI of WOT without and with respiratory gating at the EOI and EOE phases, the corresponding (b) intensity-related FPI of PV and (c) ratio-related FPI of WIR.

**Figure 8:**
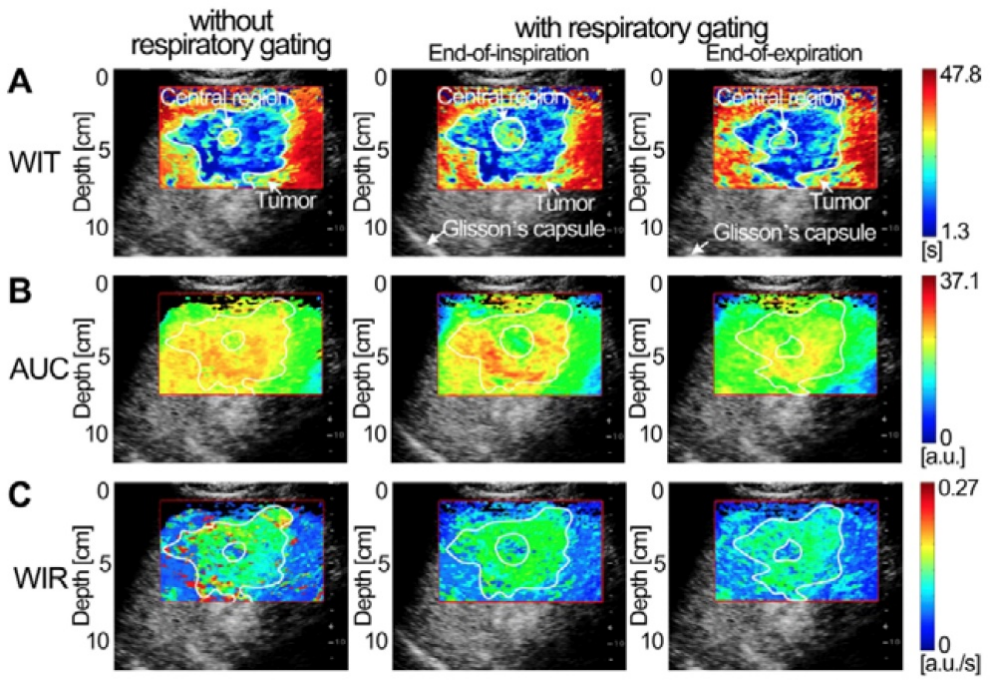
(Color online) Comparisons of* in vivo* hepatic DCEUS-based DM-FPI of a hepatic cavernous hemangioma. (a) A time-related FPI of WIT without and with respiratory gating at the EOI and EOE phases, the corresponding (b) intensity-related FPI of AUC and (c) ratio-related FPI of WIR.

**Figure 9:**
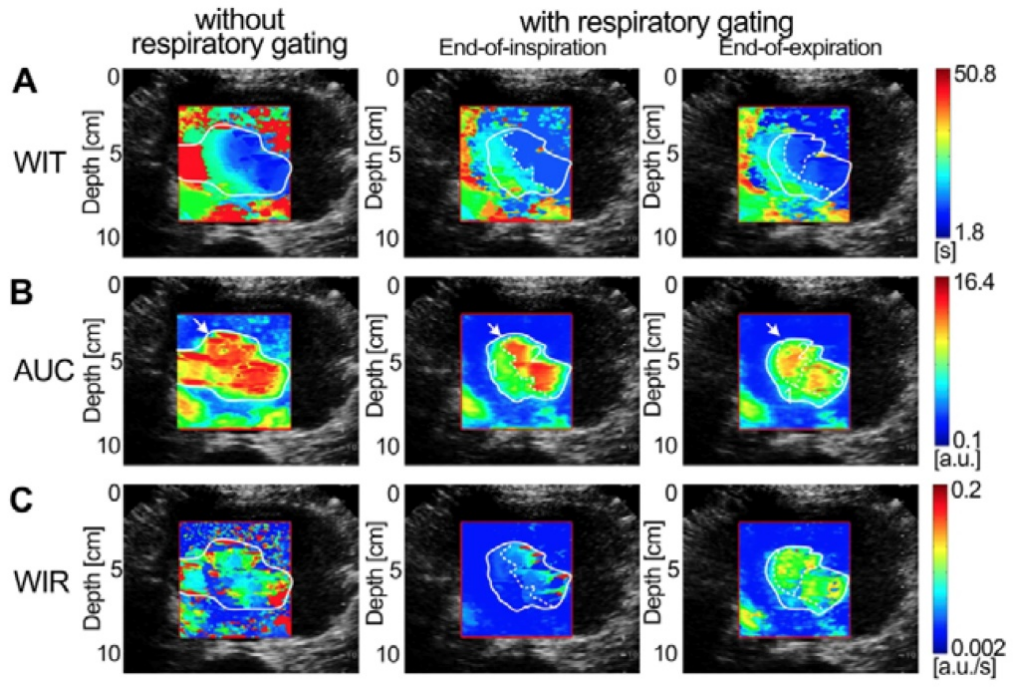
(Color online) Comparisons of* in vivo* hepatic DCEUS-based DM-FPI of a hepatocellular carcinoma. (a) A time-related FPI of WIT without and with respiratory gating at the EOI and EOE phases, the corresponding (b) intensity-related FPI of AUC and (c) ratio-related FPI of WIR.

Figures 7-9 display three groups of *in vivo* DCEUS-based dual-phase FPI_noRG_ and FPI_RG_ in the normal liver of a healthy rabbit, and the HCH and HCC of two patients, respectively. Each group of DM-FPIs included one time-related FPI of WIT or WOT, one intensity-related FPI of PV or AUC, and one ratio-related FPI of WIR. Compared with aliasing in FPI_noRG_ caused by severe out-of-plane breathing motion, *in vivo* hepatic blood velocity, perfusion charge, and rate of flow were clearly depicted by the time-, intensity-, and ratio-related FPI_RG_, respectively. Anatomical structures of the right and left hepatic veins in the normal liver were distorted in FPI_noRG_, which were recovered effectively by the dual-phase FPI_RG_ of WOT. The irregular border of the HCH was accurately identified by the dual-phase FPI_RG_ of WIT and WIR, respectively. The heterogeneous HCC angiogenesis was well quantified in the dual-phase FPI_RG_ of AUC and WIR, respectively.

**Figure 10:**
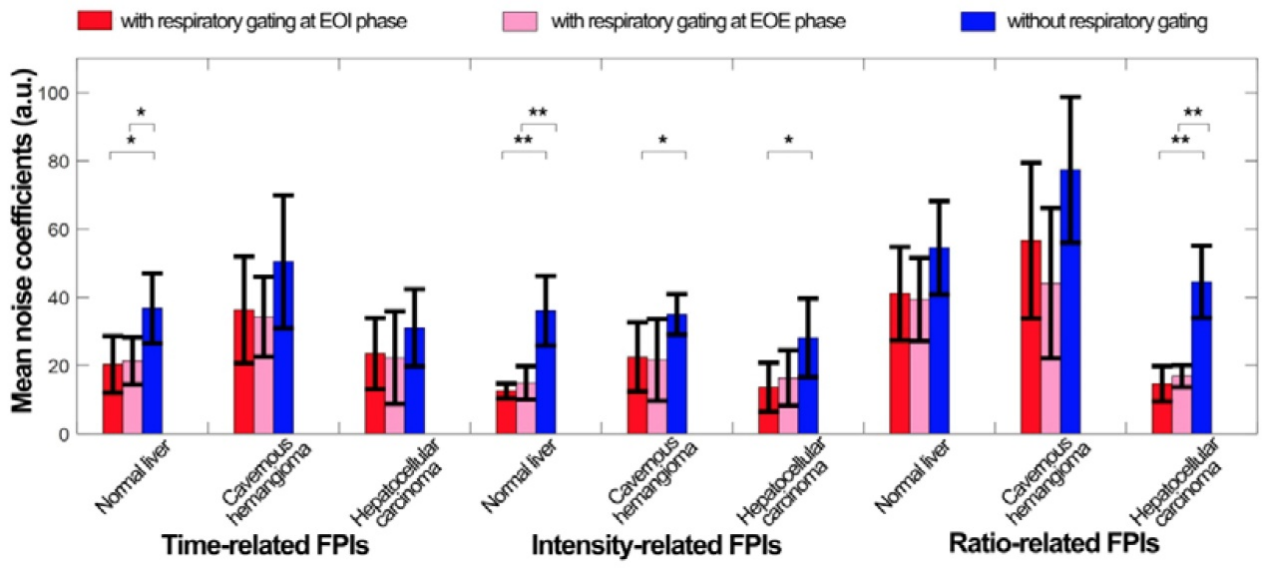
(Color online) Mean noise coefficients of *in vivo* hepatic DCEUS-based DM-FPI without and with respiratory gating using derivative PCA zero-crossing dual-phase detection.

Figure 10 quantifies the respiratory gating performance in DCEUS-based DM-FPI using MNC of *in vivo* dual-phase FPI_RG_ and FPI_noRG_. In comparison with FPI_noRG_, the decrease in MNC of time-, intensity-, and ratio-related dual-phase FPI_RG_ within the normal liver, HCH, and HCC were shown in Table 4. The corresponding MNC decreased significantly by 17.5 ± 7.1 (*p* < 0.05) in FPI_RG_. These *in vitro* and *in vivo* verifications demonstrated that the proposed DM-FPI scheme considerably removed severe out-of-plane artifacts caused by breathing motion on FPI, improved the accuracy in respiratory gating FPI. The proposed method could also characterize and distinguish the heterogeneous hemodynamics of the hepatic angiogenesis within two sections of liver cancer cells at the EOI and EOE phases.

## Discussion

### Feasibility of DCEUS-based Dual-phase Multi-parametric FPI

This study used DCEUS video loops to reconstruct DM-FPI. In comparison with radiofrequency signal, nonlinear image processing algorithms may influence DCEUS video signal 34. An impact is a possibility of losing the linear relation between the estimated and true local perfusions quantified by TICs 12, 34. However, almost all commercial ultrasound platforms do not support access to radiofrequency data, so their acquisition is impractical for clinical applications. In practice, these nonlinear effects can be suppressed under an unsaturated gain and a wide dynamic range of system settings 39. In this case, backscatter amplitudes of microbubbles are nearly proportional to their local concentration 8, 12. This is what has been done in the current study (see Table 2); high dynamic ranges were selected to collect *in vitro* and *in vivo* TICs and to guarantee the efficient reconstruction of DM-FPI 34. Additionally, due to various hepatic sampling depths in *in vitro* and *in vivo* validations and limitation of memory space in the used commercial ultrasound platforms, the frame rate and acquisition time were different in Table 2. But the major perfusion phases were collected using different transducers with similar acoustic parameter setting. Thus, the influences induced by various frame rate and acquisition time on parameter estimation and map reconstruction of AUC, WOR, and WIT were removed by normalized at the same dynamic range 8, 32 and TIC denoising methods 12, 14, 38. Considering time-consuming in TIC model fitting 12, 38, a dual-weighted moving average filter 14 was used in this study.

### Respiratory Gating using Derivative PCA Zero-crossing Dual-phase Detection

The maximum and minimum thresholds were utilized to detect respiratory phases in the estimated respiratory kinetics 19, 27, 28. In previous studies, the respiratory kinetics was estimated using the intensity threshold strategies from the fundamental mode sequence to avoid the influence of microbubble kinetics on estimated respiratory kinetics 19, 27, 28. In Figs. 2a and 2b, the two respiratory kinetic curves were estimated from the contrast mode sequence of single-mode DCEUS loops and the fundamental mode sequence of dual-mode DCEUS loops, respectively. Differences in these respiratory kinetic curves illustrated the impact of microbubble kinetics on estimated respiratory kinetics. However, the microbubble kinetics in Fig. 2a did not hamper the respiratory gating accuracy when the proposed derivative PCA zero-crossing dual-phase detection method was used. Some commercial ultrasound platforms only have the single contrast mode for DCEUS. In contrast to the intensity threshold strategy in 19, 27, 28, the derivative PCA zero-crossing dual-phase detection strategy enabled an accurate reconstruction of DM-FPI using single-mode rather than dual-mode DCEUS sequences.

### Comparison with Other Machine Learning Algorithms

Previous studies had discussed the performance of *in vitro* and* in vivo* respiratory gating or motion compensation methods based on machine learning algorithms 27, 28, 31. Mulé et al. and Gatos et al. described limitations of the negative component in PCA and the uncertain independence assumption in the independent component analysis through theoretical analyses 27, 31. However, they failed to compare the accuracy of these estimation algorithms. Our previous study quantified the accuracy and computation efficiency of independent component analysis, PCA, and non-negative matrix factorization for respiratory kinetic assessment by considering the MSE and calculation time 32. The MSE ratio of PCA versus independent component analysis was smaller by 2.7 (*p* < 0.05) and computation efficiency was quicker by 509.7 times (p < 0.01). Compared with non-negative matrix factorization, MSE of PCA was reduced by 0.3 (*p* < 0.05) and computation efficiency was improved by 20.8 times (*p* < 0.05). Thus, in comparison with independent component analysis and non-negative matrix factorization, the accuracy of estimated respiratory kinetics using PCA was improved and computation efficiency was the highest among those machine learning algorithms 32.

### Accuracy and Feasibility of DCEUS-based Dual-phase Multi-parametric FPI with Respiratory Gating

The *in vitro* validation of respiratory gating for DCEUS-based DM-FPI aimed to provide information not available on the accuracy of those previous studies 17, 27, 28 under conditions of important out-of-plane 3D respiratory motion. Given an unknown ground truth in *in vivo* experiments, only Christofides and Renault et al. evaluated the respiratory gating accuracy through ideal simulation models under in-plane and slight out-of-plane motion conditions, respectively 17, 28. However, their studies focused on the respiratory gating for DCEUS rather than FPI and did not consider severe 3D out-of-plane motion artifacts. Our previous *in vitro* studies validated the accuracy of FPI with respiratory motion compensation at in-plane condition 32 and 1D perfusion quantification with respiratory gating under out-of-plane condition 33 but severe out-of-plane motions were not considered for 2D FPI. Therefore, the respiratory gating precision of hepatic DM-FPI based on DCEUS was validated by considering multiple perspectives in this study for the first time. Moreover, under different levels of out-of-plane respiratory motions, the proposed scheme also could accurately identify the dual-phase DCEUS subsequence and well reconstruct the corresponding DCEUS-based FPI. The levels of motion artifacts were controlled by the transducer rotary displacements. Compared with FPI_noRG_, the decreases in MSE of FPI_RG_ were range from 1189.5 ± 86.71 (*p* < 0.05) to 1893.9 ± 965.4 (*p* < 0.05) under the conditions from slight to serious respiratory motion artifacts. But the increases in correlation R of FPI_RG_ were range from 0.2 ± 0.1 (*p* < 0.05) to 0.4 ± 0.2 (*p* < 0.01). These quantitative evaluations emphasized the fact that the noise level, misestimation, and various out-of-plane artifacts in FPIs induced by respiratory motion could be considerably decreased by the proposed respiratory gating DM-FPI scheme.

### Characterization of Angiogenic Heterogeneity with Dual-phase Multi-parametric FPI

HCH and HCC are respectively the most prevalent benign 40 and malignant liver tumors 1. Dual-phase FPI_RG_ clearly characterized the hepatic angiogenesis in HCH and HCC at EOI and EOE phases (Figs. 8 and 9). Although abundant HCH cells were demarcated by a fibrous capsule 40, the massive HCH (size: more than 4 cm 40) in this study clearly exhibited an undulating hemodynamic feature with an irregular border in the dual-phase FPI_RG_. Angiogenesis between the surrounding HCH and its central island of hepatocytes were also clearly characterized in the dual-phase FPI_RG_, which was consistent with histologic features of HCH in 40. Angiogenesis in HCH and HCC exhibited similar hemodynamic features with relatively smaller WIT, higher AUC, and smaller WIR in the dual-phase FPI_RG_ than those in the surrounding normal liver. Moreover, WIR and AUC in HCC angiogenesis were larger than those of HCH. As such, the angiogenesis of HCC presented a larger perfusion volume and a faster flow rate. These observations are consistent with HCH and HCC analyses but also complemented the hemodynamic analyses in 7. Indeed, our results illustrated that the dual-phase FPI_RG_ could identify tumors from the surrounding normal livers and distinguish benign HCHs from malignant HCCs.

Moreover, it is well-known that the tumor angiogenesis presents heterogeneity because of differences in cancer size, density, and architecture 1, 8, 41. The heterogeneity of the angiogenesis of HCC was more important in Fig. 9 than that of HCH in Fig. 8. The 3D spatial heterogeneity in the angiogenesis of HCC was distinctly quantified and depicted in dual-phase FPI_RG_ using various hemodynamic parameters within two anatomical sections. In comparison with the relative hypo-perfusion (*i.e*., hypoxia) zone in the No. 1 region (Fig. 9), angiogenic hyper-perfusion (*i.e*., hyperoxia) zones were characterized in the No. 2 region in FPI_RG_ at the EOI phase and in the No. 2 and No. 3 regions in FPI_RG_ at the EOE phase. The heterogeneous angiogenic hemodynamics in HCC and HCH were visualized and quantified using dual-phase FPI_RG_. Additionally, angiogenesis not only is an effective indicator to diagnose cancer but also becomes one of the principal targets of several antiangiogenic therapies 2, 3, 42. Therefore, the proposed DM-FPI scheme is a potential method to identify hepatic tumors, evaluate therapeutic effectiveness, adjust drug doses, and modify treatments if necessary.

### Comparison with Other Quantitative DCEUS and Functional Imaging Techniques

Several quantitative DCEUS techniques have been developed to directly quantify the microvascular hemodynamics. 1D TIC analysis techniques were proposed to qualitatively indicate perfusion differences within normal and lesion tissues, which were further developed to estimate single 43 and multi-parameters 44 to quantify perfusion differences. Goetti et al. and Zhang et al. proposed the perfusion classification method 16 and TIC-based factor analysis method 7 to characterize differences between benign and malignant focal lesions, respectively. Kuenen et al. 12 and Greis et al. 45 proposed 2D multi-parametric FPI to respectively quantify renal and hepatic microvascular hemodynamic distributions. Currently, researchers are focusing on improving the accuracy and robustness of FPI techniques and new clinical applications were proposed 8, 16. The resolution and contrast of FPI for intratumoral angiogenesis were improved 8, 20. Artifacts induced by slight out-of-plane motion were overcome for the abdominal DCEUS-based FPI in our previous studies 19, 32. A 2D matrix transducer was used to produce 3D DCEUS 6 and volumetric perfusion parameters were then calculated to assess the antiangiogenic treatments 46, which overcame limitations of conventional quantitative DCEUS techniques without 3D perfusion information. However, these new 3D quantitative DCEUS techniques are limited by 1D volumetric data evaluation, the lack of 2D assessment of hemodynamic distribution, the serious alteration caused by breathing motion, and by the expensive ultrasound platforms required to produce those images 6, 46.

Moreover, other FPI approaches on account of nuclear magnetic spin imaging and computerized tomography are also widely used to diagnose and stage tumors and to monitor their response to treatments 47-49. By contrast, these functional imaging techniques are limited by low time resolution and also disturbed by motion artifacts 48, 49. The DCEUS-based FPI has the advantages of ultrasound imaging mentioned in the Introduction 10-12 but is also limited by low reproducibility and lack of standard scanning section due to artifacts of the sonographer. In a sense, these limitations in DCEUS-based FPI might be known as the better portability and flexibility. Compared with 1D, 2D, and 3D quantitative DCEUS methods and other functional imaging techniques, the proposed DM-FPI scheme has three important advanced features: the fully automatic respiratory gating, the dual-phase mode, the possibility of performing multi-parametric reconstructions under severe conditions of out-of-plane 3D respiratory motions. Thus, the proposed respiratory gating strategy, using PCA combined with derivative zero-crossing phase detection, made it possible to accurately identify the dual-phase DCEUS subsequences and to depict the hepatic hemodynamics within multi-anatomical sections. In a sense, the proposed DM-FPI scheme might balance the conventional known 2D and 3D quantitative DCEUS techniques and might be described as a 2.5D FPI technique. However, the detailed perfusion information between EOI and EOE phases was missed in the proposed scheme. A 3D respiratory gating FPI might overcome this limitation and its feasibility should be further studied at a bigger database in the further.

## Conclusion

This study proposed a hepatic respiratory gating DM-FPI technique based on DCEUS utilizing derivative PCA. Accuracy and feasibility of the proposed technique were respectively illustrated through *in vitro* and *in vivo* hepatic perfusion validations under conditions of severe out-of-plane respiratory motions. Compared with approaches without respiratory gating, the proposed DM-FPI technique significantly removed out-of-plane artifacts and parametric misestimation induced by respiratory kinetics and improved the respiratory gating accuracy and robustness. Respiratory gating for DM-FPI could characterize and distinguish heterogeneous angiogenic hemodynamics within two sections in normal livers and in benign and malignant liver tumors. The proposed scheme may assist clinicians to identify benign and malignant hepatic tumors, characterize tumor grading and staging, evaluate therapeutic effectiveness, and guide appropriate therapies for liver cancers.

## Figures and Tables

**Table 1:** List of abbreviations

DCEUS	dynamic contrast-enhanced ultrasound	HCH	hepatic cavernous hemangioma
PCA	principal component analysis	HCC	hepatocellular carcinoma
TIC	time-intensity curve	WIT	wash-in time
TIC_RG_	TIC with respiratory gating	WOT	wash-out time
TIC_noRG_	TIC without respiratory gating	PV	peak value
TIC_static_	static TIC without respiratory motion artifacts	AUC	area under curve
FPI	functional perfusion imaging	WIR	wash-in rate
FPI_RG_	FPI with respiratory gating	WOR	wash-out rate
FPI_noRG_	FPI without respiratory gating	SCR	signal-to-clutter ratio
FPI_static_	static FPI without respiratory motion artifacts	MSE	mean square error
DM-FPI	dual-phase multi-parametric FPI	R	correlation coefficient
EOI	end-of-inspiration	MNC	mean noise coefficient
EOE	end-of-expiration		

**Table 2:** *In vitro* and *in vivo* experimental parameter settings

	*In vitro* model	*In vivo* liver		
		Normal^†^	HCH	HCC
Respiratory number	889	216	51	60
Mechanical index	0.05	0.05	0.07	0.10
Work frequency (MHz)	4	4.4	4.4	4.4
Frame rate (Hz)	21.3	15	10	30
Dynamic range (dB)	154	120	135	135
Valid perfusion time (s)	120-160	90-135	60-90	60-90

† normal hepatic perfusion experiments of healthy rabbits.

**Table 3:** Decrease in MSE and increase in correlation R of *in vitro* FPI_RG_ compared with FPI_noRG_

	FPI	Decrease in MSE	Increase in R
EOI phase	Time-related	2418.6 ± 257.4**	0.4 ± 0.1**
	Intensity-related	2653.4 ± 49.9**	0.6 ± 0.2**
	Ratio-related	2063.5 ± 736.6**	0.6 ± 0.1**
EOE phase	Time-related	417.2 ± 717.5	0.3 ± 0.1**
	Intensity-related	1011.3 ± 578.4*	0.4 ± 0.2*
	Ratio-related	2799.6 ± 1116.7**	0.2 ± 0.1

* *p*< 0.05, and ** *p* < 0.01.

**Table 4:** Decrease in MNC of *in vivo* FPI_RG_ compared with FPI_noRG_

	FPI	Normal^†^	HCH	HCC
EOI phase	Time-related	16.4 ± 9.3*	14.1 ± 13.6	7.5 ± 8.8
	Intensity-related	23.5 ± 6.2**	12.5 ± 8.0*	14.5 ± 9.4*
	Ratio-related	13.4 ± 10.7	20.7 ± 18.1	29.9 ± 7.9**
EOE phase	Time-related	15.4 ± 8.6*	16.2 ± 15.6	8.8 ± 11.4
	Intensity-related	21.3 ± 7.6**	13.4 ± 9.0	11.8 ± 7.8
	Ratio-related	15.1 ± 12.9	33.3 ± 20.7	27.7 ± 6.9**

† normal hepatic perfusion experiments of healthy rabbits, * *p*< 0.05, and ** *p* < 0.01.
